# Supplementary material for: End Sequence Analysis Toolkit (ESAT) expands the extractable information from single-cell RNA-seq data
Source: Genome Res. 2016 Oct;26(10):1397–410. doi: 10.1101/gr.207902.116 (PMC5052061; doi:10.1101/gr.207902.116)
Supplement: Supplemental Material [file supp_gr.207902.116_Supp_Materials.tar.gz › ESAT-esat_v0.1/doc/html/classjaligner_1_1_sequence.html]

ESAT: jaligner.Sequence Class Reference


ESAT


- Main Page
- Related Pages
- Packages
- Classes
- Files

- Class List
- Class Index
- Class Hierarchy
- Class Members

All Classes Namespaces Files Functions Variables Enumerator Pages

- jaligner
- Sequence

Public Member Functions |
Static Public Attributes |
List of all members

jaligner.Sequence Class Reference

Inheritance diagram for jaligner.Sequence:

[legend]

Collaboration diagram for jaligner.Sequence:

[legend]

|  |  |
| --- | --- |
| Public Member Functions | |
|  | Sequence () |
|  | |
|  | Sequence (String sequence) |
|  | |
|  | Sequence (String name, char[] sequence) |
|  | |
|  | Sequence (String name, String sequence) |
|  | |
|  | Sequence (String sequence, String id, String description, int type) |
|  | |
| String | getSequence () |
|  | |
| void | setSequence (String sequence) |
|  | |
| String | getId () |
|  | |
| void | setId (String id) |
|  | |
| String | getDescription () |
|  | |
| void | setDescription (String description) |
|  | |
| int | getType () |
|  | |
| void | setType (int type) |
|  | |
| int | length () |
|  | |
| String | subsequence (int index, int length) |
|  | |
| char | acidAt (int index) |
|  | |
| char[] | toArray () |
|  | |
| String | toString () |
|  | |

|  |  |
| --- | --- |
| Static Public Attributes | |
| static final int | NUCLEIC = 0 |
|  | |
| static final int | PROTEIN = 1 |
|  | |

## Detailed Description

A basic (nucleic or protein) sequence. It's a wrapper to java.lang.String.

Author
:   Ahmed Moustafa (ahmed.nosp@m.@use.nosp@m.rs.sf.nosp@m..net)

## Constructor & Destructor Documentation

|  |  |  |  |  |
| --- | --- | --- | --- | --- |
| jaligner.Sequence.Sequence | ( |  | ) |  |

Constructor

|  |  |  |  |  |  |
| --- | --- | --- | --- | --- | --- |
| jaligner.Sequence.Sequence | ( | String | *sequence* | ) |  |

Constructor

Parameters
:   |  |  |
    | --- | --- |
    | sequence |  |

|  |  |  |  |
| --- | --- | --- | --- |
| jaligner.Sequence.Sequence | ( | String | *name*, |
|  |  | char[] | *sequence* |
|  | ) |  |  |

Constructor

Parameters
:   |  |  |
    | --- | --- |
    | name |  |
    | sequence |  |

|  |  |  |  |
| --- | --- | --- | --- |
| jaligner.Sequence.Sequence | ( | String | *name*, |
|  |  | String | *sequence* |
|  | ) |  |  |

Constructor

Parameters
:   |  |  |
    | --- | --- |
    | name |  |
    | sequence |  |

|  |  |  |  |
| --- | --- | --- | --- |
| jaligner.Sequence.Sequence | ( | String | *sequence*, |
|  |  | String | *id*, |
|  |  | String | *description*, |
|  |  | int | *type* |
|  | ) |  |  |

Constructor

Parameters
:   |  |  |
    | --- | --- |
    | sequence |  |
    | id |  |
    | description |  |
    | type |  |

## Member Function Documentation

|  |  |  |  |  |  |
| --- | --- | --- | --- | --- | --- |
| char jaligner.Sequence.acidAt | ( | int | *index* | ) |  |

Returns the acid at specific location in the sequence

Parameters
:   |  |  |
    | --- | --- |
    | index |  |

Returns
:   acid at index

|  |  |  |  |  |
| --- | --- | --- | --- | --- |
| String jaligner.Sequence.getDescription | ( |  | ) |  |

Returns the sequence description

Returns
:   Returns the description

|  |  |  |  |  |
| --- | --- | --- | --- | --- |
| String jaligner.Sequence.getId | ( |  | ) |  |

Returns the sequence id

Returns
:   Returns the id

Here is the caller graph for this function:

|  |  |  |  |  |
| --- | --- | --- | --- | --- |
| String jaligner.Sequence.getSequence | ( |  | ) |  |

Returns the sequence string

Returns
:   Returns the sequence

|  |  |  |  |  |
| --- | --- | --- | --- | --- |
| int jaligner.Sequence.getType | ( |  | ) |  |

Returns the sequence type (nucleic or protein)

Returns
:   Returns the type

|  |  |  |  |  |
| --- | --- | --- | --- | --- |
| int jaligner.Sequence.length | ( |  | ) |  |

Returns the length of the sequence

Returns
:   sequence length

Here is the caller graph for this function:

|  |  |  |  |  |  |
| --- | --- | --- | --- | --- | --- |
| void jaligner.Sequence.setDescription | ( | String | *description* | ) |  |

Sets the sequence description

Parameters
:   |  |  |
    | --- | --- |
    | description | The description to set |

|  |  |  |  |  |  |
| --- | --- | --- | --- | --- | --- |
| void jaligner.Sequence.setId | ( | String | *id* | ) |  |

Sets the sequence id

Parameters
:   |  |  |
    | --- | --- |
    | id | The id to set |

|  |  |  |  |  |  |
| --- | --- | --- | --- | --- | --- |
| void jaligner.Sequence.setSequence | ( | String | *sequence* | ) |  |

Sets the sequence string

Parameters
:   |  |  |
    | --- | --- |
    | sequence | The sequence to set |

|  |  |  |  |  |  |
| --- | --- | --- | --- | --- | --- |
| void jaligner.Sequence.setType | ( | int | *type* | ) |  |

Sets the sequence type (nucleic or protein)

Parameters
:   |  |  |
    | --- | --- |
    | type | The type to set |

|  |  |  |  |
| --- | --- | --- | --- |
| String jaligner.Sequence.subsequence | ( | int | *index*, |
|  |  | int | *length* |
|  | ) |  |  |

Returns a subsequence

Parameters
:   |  |  |
    | --- | --- |
    | index | start index |
    | length | length of subsequence |

Returns
:   subsequence

Here is the call graph for this function:

|  |  |  |  |  |
| --- | --- | --- | --- | --- |
| char [] jaligner.Sequence.toArray | ( |  | ) |  |

Returns the sequence as an array of characters.

Returns
:   array of chars.

|  |  |  |  |  |
| --- | --- | --- | --- | --- |
| String jaligner.Sequence.toString | ( |  | ) |  |

Returns the sequence id and the sequence string

Returns
:   Returns the sequence id and the sequence string

## Member Data Documentation

final int jaligner.Sequence.NUCLEIC = 0

static

Sequence type nucleic.

final int jaligner.Sequence.PROTEIN = 1

static

Sequence type protein.

---

The documentation for this class was generated from the following file:

- java/jaligner/Sequence.java


---

Generated on Fri Aug 15 2014 15:11:05 for ESAT by  

 1.8.7
